# Patient perception regarding privacy and confidentiality: A study from the emergency department of a tertiary care hospital in Karachi, Pakistan

**DOI:** 10.12669/pjms.38.ICON-2022.5785

**Published:** 2022-01

**Authors:** Syed Ghazanfar Saleem, Saima Ali, Nida Ghouri, Quratulain Maroof, Muhammad Imran Jamal, Tariq Aziz, David Shapiro, Megan Rybarczyk

**Affiliations:** 1 Dr. Syed Ghazanfar Saleem, MBBS, FCPS, Department of Emergency Medicine, The Indus Hospital; Karachi, Pakistan; 2 Saima Ali, MBBS, FCPS, Department of Emergency Medicine, The Indus Hospital; Karachi, Pakistan; 3 Nida Ghouri, Indus Hospital Research Center, The Indus Hospital and Health Network, Karachi, Pakistan; 4 Quratulain Maroof, Department of Emergency Medicine, The Indus Hospital; Karachi, Pakistan; 5 Muhammad Imran Jamal, Department of Emergency Medicine, The Indus Hospital; Karachi, Pakistan; 6 Tariq Aziz, Department of Emergency Medicine, The Indus Hospital; Karachi, Pakistan; 7 David Shapiro, MD, Department of Emergency Medicine, University of Pennsylvania; Philadelphia, Pennsylvania, USA; 8 Megan Rybarczyk, MD, MPH, FACEP, Department of Emergency Medicine, University of Pennsylvania; Philadelphia, Pennsylvania, USA

**Keywords:** Emergency Department, Privacy, Confidentiality, Survey, Patient perception, Patient-provider interactions

## Abstract

**Background and Objective:**

Maintaining privacy and ensuring confidentiality with patients is paramount to developing an effective patient-provider relationship. This is often challenging in over-crowded Emergency Departments (EDs). This survey was designed to explore patients’ perceptions on maintenance of privacy and confidentiality and their subsequent interactions with providers in a busy tertiary care hospital in Karachi.

**Methods:**

Trained nursing staff conducted structured interviews with 571 patients who presented to The Indus Hospital (TIH) ED from January to December 2020. All patients were 14 years of age or older, could speak and understand Urdu, and provide informed consent. Patients were asked about their perceptions of privacy and confidentiality in the ED and whether this affected their interactions with providers.

**Results:**

Respondents were primarily men (64%) under the age of 45 (62%) presenting for the first time (49%). The majority of patients felt that privacy and confidentiality were maintained, however 10% of patients reported that they had rejected examination due to privacy concerns and 15% of patients reported that they had changed or omitted information provided to a provider due to confidentiality concerns. There was correlation between privacy and confidentiality concerns and patient-provider interactions (p<0.0001).

**Conclusions:**

Despite the often over-crowded and busy environment of the ED, patients generally felt that privacy and confidentiality were maintained. Given the correlation between perception and behavior and the importance of an effective patient-provider relationship, particularly in the acute setting when morbidity and mortality is high, initiatives that focus on maintaining privacy and confidentiality should be pursued.

## INTRODUCTION

The concept of privacy emphasizes the individuality of a person and concerns a human being’s decision to deny or grant access to self and individual behaviors, opinions, and attitudes; to personal or identifying information; and to private property or territory. More broadly, privacy includes physical seclusion, protection of personal information, protection of identity, and the ability to make choices without interference. In short, it is the right of an individual to selective expression of themselves, to sharing information about themselves, and to making decisions that affect them personally without interference.^1^

Confidentiality refers to protection of personal information. In medicine, confidentiality acknowledges respect for privacy, decreases vulnerability, and ensures trust – all of which are imperative for obtaining an accurate history and exam, and, ultimately, diagnosis.^2^,^3^

Physical and environmental limitations as well as high patient volumes make the protection of patient privacy and confidentiality challenging in the Emergency Department (ED) setting.^2^ The spaces for patients can be undersized; patients may be placed in close proximity to each other, family members, health care providers, and staff when volume is high; there may not be solid or enclosed spaces; and health care providers are often moving quickly between spaces.^2^,^3^ For example, with ED overcrowding, patients are often placed in or near hallways, exacerbating the challenges of protecting privacy and ensuring confidentiality.^4^ Furthermore, patients in the ED often have sensitive issues related to their disease and livelihood, such as substance use disorders, intimate partner violence, concerns about sexual and reproductive health, and psychiatric conditions. Additionally, the privacy and confidentiality of severely ill or injured patients is often not a focus during acute resuscitation, and patients in this state may also not be capable of advocating for themselves.^5^,^6^ As a result, the responsibility lies with ED providers to be sensitive to issues concerning privacy and confidentiality in order to establish an effective patient-physician relationship, to foster an environment where patients can disclose sensitive and essential information, and be able to detect and manage acute illness and injury that might otherwise result in high morbidity or mortality.^7^-^10^ Furthermore, privacy and confidentiality are one of the core indicators of patients’ satisfaction and quality care.^11^

The Indus Hospital (TIH) Korangi campus is a 300-bed tertiary care hospital in one of the lowest resourced areas of Karachi, one of the largest cities in the world. TIH is one of the main hospitals of the Indus Hospital and Health Network (IHHN), a private health network that provides care free of cost through a network of public outreach programs, clinics, physical rehabilitation centers, blood centers, and hospitals throughout all of the administrative units of Pakistan. To date, there is no information about patients’ perspectives of the protection of their privacy and confidentiality as it relates to their interaction with providers and overall satisfaction with care at TIH. This study investigates patients’ perceptions of maintenance of privacy and protection of confidentiality as well as potential correlations with alterations in patient-provider interactions during their ED visit.

## METHODS

A prospective convenience sample of patients at TIH, a single tertiary care level center in Karachi, Pakistan, was surveyed. Ten nursing staff working on different shifts received standardized training from study leadership on conducting the structured interview, and they conducted interviews for approximately two hours daily during the study period of January to December 2020. Patients above 14 years of age who were able to understand and speak Urdu, with a triage coding of P3 or P4 (Manchester Triage System), and who presented to the Adult ED were included.^12^ Patients with a triage level of P1, P2, or P5; patients brought dead/expired while in the ED; patients with altered mental status; patients unable to understand or speak Urdu, and patients who did not provide consent were excluded. Verbal consent was obtained from study participants. The study was approved by the IRB (IRD_IRB_2019_09_015).

Data was analyzed using Microsoft Excel (Microsoft 365, Redmond, Washington) and Stata (StataCorp, release 17.0 BE, College Station, Texas). Spearman’s rank correlation was used to investigate for a potential correlation between the patient’s perceptions and the impact on patient-provider interactions, and the Wilcoxon rank-sum test was used to identify differences in responses between demographic groups.

## RESULTS

Overall, 571 interviews meeting the pre-specified inclusion criteria were conducted. The average age of participants was 41 years (range 14-85), with 366 male patients, 204 female patients, and one transgender patient. Demographic information of the respondents is summarized in Table-I. Primary presenting chief concerns are summarized by general categories in Table-II.

**Table I:** Demographic information of respondents.

Variable	Detail: n (%)
Age	14-25: 113 (20%)
	26-35: 122 (21%)
	36-45: 120 (21%)
	46-55: 104 (18%)
	56-65: 72 (13%)
	65+: 40 (7%)
Gender	Women: 204 (36%)
	Men: 366 (64%)
	Transgender: 1 (<1%)
Primary Language	Urdu: 270 (47%)
	Punjabi: 88 (15%)
	Pashto: 58 (10%)
	Sindhi: 54 (9%)
	Other: 90 (16%)
	Not Specified: 11 (2%)
Education	None: 282 (50%)
	Formal: 288 (50%); mean 10 years (range: 1-25)
	Not Specified: 1 (<1%)
Triage Category	P3: 512 (90%)
	P4: 59 (10%)
Time of Presentation	Morning (6AM to 11:59AM): 242 (42%)
	Afternoon (12PM to 5:59PM): 205 (36%)
	Evening (6PM to 11:59PM): 92 (16%)
	Night (12AM to 5:59AM): 32 (6%)
ED LOS	≤6 hours: 309 (54%)
	>6 to 12 hours: 218 (38%)
	>12 hours: 44 (8%)
Frequency of ED Presentation	First presentation: 279 (49%)
	Once a year: 80 (14%)
	Once a month to once every 6 months: 122 (21%)
	Once a month: 90 (16%)

ED – Emergency Department; LOS – Length of Stay.

**Table II:** Primary chief concerns of the respondents.

Presenting Symptom	Percent
Gastrointestinal Symptoms	30%
Genitourinary Symptoms	12%
Trauma	12%
Chest Pain/Cardiac Symptoms	10%
Musculoskeletal Symptoms	10%
Fever	7%
Respiratory Symptoms	6%
Headache/Neurologic Symptoms	3%
Other	10%

We first evaluated patient’s perceptions of confidentiality overall. 54% of patients “Agreed” or “Strongly Agreed” that the confidentiality of their medical information was properly maintained (Fig.1A), and 55% “Agreed” or “Strongly Agreed” that patient information was not kept open in front of other patients.

**Fig.1A:**
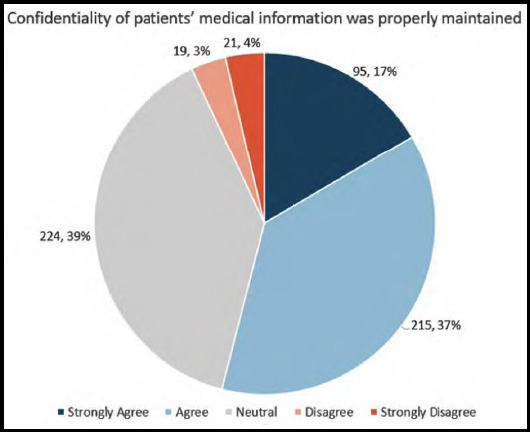
Patient perception of maintenance of confidentiality of medical information.

We next evaluated whether patients felt privacy was maintained during their clinical evaluation. 76% “Strongly Agreed” that they were given enough privacy when discussing their medical conditions (Fig.1B), and 79% “Agreed” or “Strongly Agreed” that other patients could not hear their conversations with health care providers. Additionally, 83% “Agreed” or “Strongly Agreed” that their personal information could not be heard by other people, while 79% “Agreed” or “Strongly Agreed” that they had not heard the conversations of others. Furthermore, 70% of patients “Strongly Agreed” that there was a screen or curtain around the bed to ensure privacy, 80% of patients “Disagreed” or “Strongly Disagreed” that unauthorized persons had been able to see them while they were receiving assistance and that people not attending to them had been able to see intimate parts of their body while receiving attention. Finally, 85% of patients “Disagreed” or “Strongly Disagreed” that they had been able to see other patients while they were being examined.

**Fig.1B:**
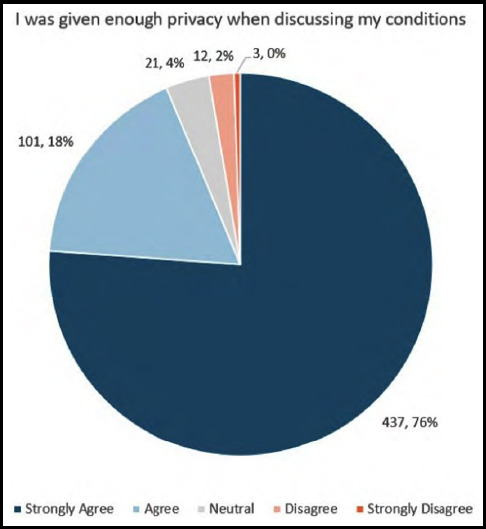
Patient’s perception of privacy with speaking about their concerns.

Finally, we evaluated the effects of concerns about privacy and/or confidentiality on the patient-provider interaction. About 85% of patients “Disagreed” or “Strongly Disagreed” that they had changed or omitted information given to health care providers because they felt it could be heard (Fig.2A), and 90% of patients “Disagreed” or “Strongly Disagreed” that they had rejected physical examination because they thought they could be seen by unauthorized persons (Fig.2B). Conversely, 15% of patients were “Neutral”, “Agreed”, or “Strongly Agreed” that they had changed or omitted information, and 10% were “Neutral”, “Agreed”, or “Strongly Agreed” that they had rejected physical examination by a doctor because of privacy concerns. There was a correlation between patient concerns about protection of privacy and/or confidentiality and a negative impact on the patient-provider interaction (Spearman’s rho 0.3121, p<0.0001).

**Fig.2A:**
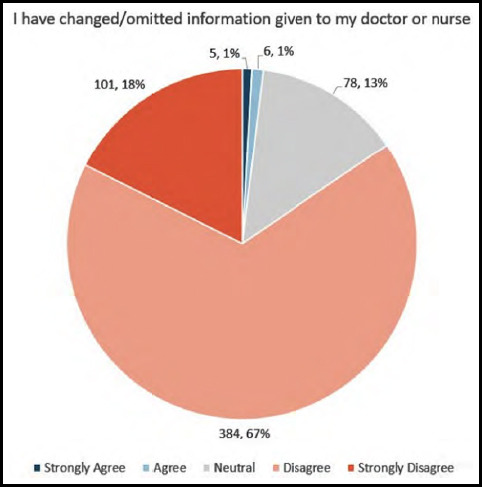
Patient-provider interactions regarding communication as a result of perceived lack of maintenance of privacy and/or confidentiality.

**Fig.2B:**
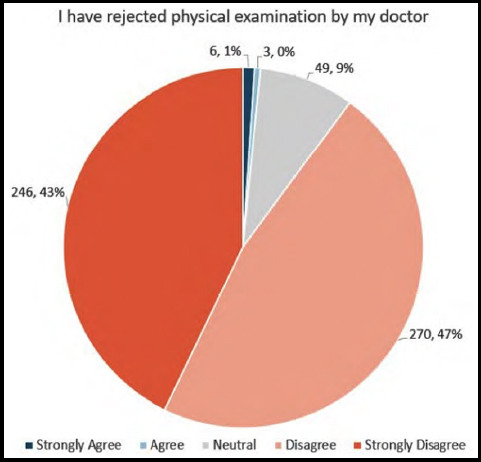
Patient-provider interactions regarding physical examination as a result of perceived lack of maintenance of privacy and/or confidentiality.

Finally, there were significant differences noted on some of the responses to some of the questions both on perception (e.g. “confidentiality of patient’s medical information was properly maintained”; z = 1.977, p = 0.05) and interaction with staff (e.g. “I have rejected physical examination by my doctor because I had feeling the process could be seen by unauthorized persons”; z = -3.456, p=0.0005), with more women agreeing/strongly agreeing with the former statement and fewer women strongly disagreeing with the latter statement.

## DISCUSSION

Privacy and confidentiality are cornerstones of an effective patient-provider relationship built on trust, particularly in an acute setting when morbidity and mortality risks are high. Overall, in this study, patients agreed that their privacy and confidentiality were maintained in a busy, tertiary center level ED in Karachi. The survey did also reveal, however, that patient perceptions may impact patient-physician interactions. This is consistent with prior work also demonstrating the correlation between perception of privacy and its effects on care.^13^-^15^

More broadly, this work is the first step in identifying and addressing patient concerns about privacy in the ED as well as further investigation of potential differences in concerns among different patient populations (e.g., between men and women). Understanding patient concerns and recognizing the impact they have on patient care is fundamental to improving patient experience and patient outcomes, and targeted interventions to increase the protection of privacy and confidentiality have been shown to be effective in the ED setting.^16^,^17^

### Limitations of the study:

It include potential selection bias for more satisfied patients given the reliance on convenience sampling. Additionally, response bias is a potential confounding factor, particularly as interviewers were nursing staff and not research assistants or other staff removed from patient care. Although all respondents could speak and understand Urdu, it was only the primary language in 47% of respondents, so language barriers may have complicated the assessments. Finally, interviewer bias is also a factor, although this was minimized by consistent training of interviewers.

Despite these limitations, this survey highlights a large sample population from a diverse and busy ED in Karachi. We demonstrate that, while patients report satisfaction with privacy and confidentiality in the ED in general, their concerns do affect the patient-provider relationship and clinical care; therefore, targeted interventions to address confidentiality may help not only improve patient satisfaction, but also outcomes.

## CONCLUSION

Despite the over-crowded and busy environment of the ED, patients generally felt that privacy and confidentiality was maintained. Given the correlation between perception and behavior, and the importance of an effective patient-provider relationship, particularly in the acute setting when morbidity and mortality is high, initiatives that focus on maintaining privacy and confidentiality should be pursued.

### Authors’ Contribution:

**SGS, SA, NG, QM, MIJ, and TA** conceived, designed, and did data collection.

**NG, DS, and MR** did data analysis.

**MR** did manuscript writing.

**SGS, SA, NG, QM, MIJ, TA, DS, and MR** reviewed the manuscript.

**SGS, SA, and MR** did final review and approval of the manuscript.
